# A transcriptomic study for identifying cardia‐ and non–cardia‐specific gastric cancer prognostic factors using genetic algorithm‐based methods

**DOI:** 10.1111/jcmm.15618

**Published:** 2020-07-10

**Authors:** Junyi Xin, Yanling Wu, Xiaowei Wang, Shuwei Li, Haiyan Chu, Meilin Wang, Mulong Du, Zhengdong Zhang

**Affiliations:** ^1^ Department of Environmental Genomics Jiangsu Key Laboratory of Cancer Biomarkers, Prevention and Treatment Collaborative Innovation Center for Cancer Personalized Medicine School of Public Health Nanjing Medical University Nanjing China; ^2^ Department of Genetic Toxicology The Key Laboratory of Modern Toxicology of Ministry of Education Center for Global Health School of Public Health Nanjing Medical University Nanjing China; ^3^ Department of Biostatistics Center for Global Health School of Public Health Nanjing Medical University Nanjing China

**Keywords:** biomarker, gastric cancer, genetic algorithm, prognosis, site specificity

## Abstract

Gastric cancer (GC) is a heterogeneous tumour with numerous differences of epidemiologic and clinicopathologic features between cardia cancer and non‐cardia cancer. However, few studies were performed to construct site‐specific GC prognostic models. In this study, we identified site‐specific GC transcriptomic prognostic biomarkers using genetic algorithm (GA)‐based support vector machine (GA‐SVM) and GA‐based Cox regression method (GA‐Cox) in the Cancer Genome Atlas (TCGA) database. The area under time‐dependent receive operating characteristic (ROC) curve (AUC) regarding 5‐year survival and concordance index (C‐index) was used to evaluate the predictive ability of Cox regression models. Finally, we identified 10 and 13 prognostic biomarkers for cardia cancer and non‐cardia cancer, respectively. Compared to traditional models, the addition of these site‐specific biomarkers could notably improve the model preference (cardia: AUC_traditional_ vs AUC_combined_ = 0.720 vs 0.899, *P* = 8.75E‐08; non‐cardia: AUC_traditional_ vs AUC_combined_ = 0.798 vs 0.994, *P* = 7.11E‐16). The combined nomograms exhibited superior performance in cardia and non‐cardia GC survival prediction (C‐index_cardia_ = 0.816; C‐index_noncardia_ = 0.812). We also constructed a user‐friendly GC site‐specific molecular system (GC‐SMS, https://njmu‐zhanglab.shinyapps.io/gc_sms/), which is freely available for users. In conclusion, we developed site‐specific GC prognostic models for predicting cardia cancer and non‐cardia cancer survival, providing more support for the individualized therapy of GC patients.

## 1 INTRODUCTION

Gastric cancer (GC) is the fifth most common cancer and the third leading cause of cancer death worldwide, with estimated 1.03 million new cases and 0.78 million deaths in 2018.^1^ Based on pathogenic site, GC can be classified into cardia cancer and non‐cardia cancer. To date, amounting studies have demonstrated that GC is a heterogeneous tumour with numerous differences of epidemiologic and clinicopathologic features between cardia cancer and non‐cardia cancer.^2^, ^3^

It is well known that GC patients have a poor prognosis with a 5‐year overall survival rate <40%.^4^ Meanwhile, several studies have found that the survival rate of cardia cancer patients was significantly lower than that of non‐cardia cancer patients, indicating the diverse prognosis between cardia cancer and non‐cardia cancer.^5^, ^6^ Besides, growing evidence has revealed that, in addition to clinical factors (eg age and clinical stage), genetic factors (eg genetic variants and genes expression level) may play important roles in GC survival prediction.^7^, ^8^ Therefore, it is required to find potential site‐specific biomarkers that can be used to individually predict cardia and non‐cardia GC prognosis.

Recently, with the development of high‐throughput biotechnology, how to perform feature selection in high‐dimensional data with relatively small sample size has been a great challenge. Genetic algorithm (GA), a searching algorithm based on natural selection, crossover and mutation, has been reported to be a very efficient method for feature selection.^9^ Several studies have demonstrated that GA‐based features selection methods can significantly improve the predictive accuracy of diseases risk prediction models.^10^, ^11^

In this study, to identify potential cardia‐ and non–cardia‐specific GC prognostic biomarkers, we performed a comprehensive analysis using GA‐based support vector machine (GA‐SVM) and GA‐based Cox regression method (GA‐Cox) in the Cancer Genome Atlas (TCGA) stomach adenocarcinoma (STAD) transcriptomic data.

## 2 MATERIALS AND METHODS

### 2.1 Data collection

We downloaded GC transcriptomic RNA sequence data with clear definitions of tumour origin from TCGA STAD database (October 30, 2018), including gene expression data sets (fragments per kilobase of transcript per million mapped reads [FPKM], 359 GC tumour tissues and 32 normal tissues), lncRNA expression datasets (FPKM, 359 GC tumour tissues and 32 normal tissues) and miRNA expression data sets (reads per million [RPM], 417 GC tumour tissues and 41 normal tissues) for analysis. Furthermore, a total of 87 cardia and 264 non‐cardia cancer patients with complete transcriptomic data and follow‐up information were remained for comprehensive survival analysis.

### 2.2 Site‐specific biomarkers identification

The TCGA STAD data were firstly normalized by log_2_ (*x* + 1) transformed. We separately used unpaired Student's *t* test to perform differential expression analysis in cardia and non‐cardia cancers, and extracted site‐specific biomarkers based on the following criteria: (a) call rate (percent of biomarkers with expression value >0) >70%; (b) |log_2_(fold change [FC])| > 1; (c) *P* value for Student's *t* test < 0.05; and (d) *P* value for univariate Cox test < 0.05.

### 2.3 GA‐SVM

**FIGURE 1:**
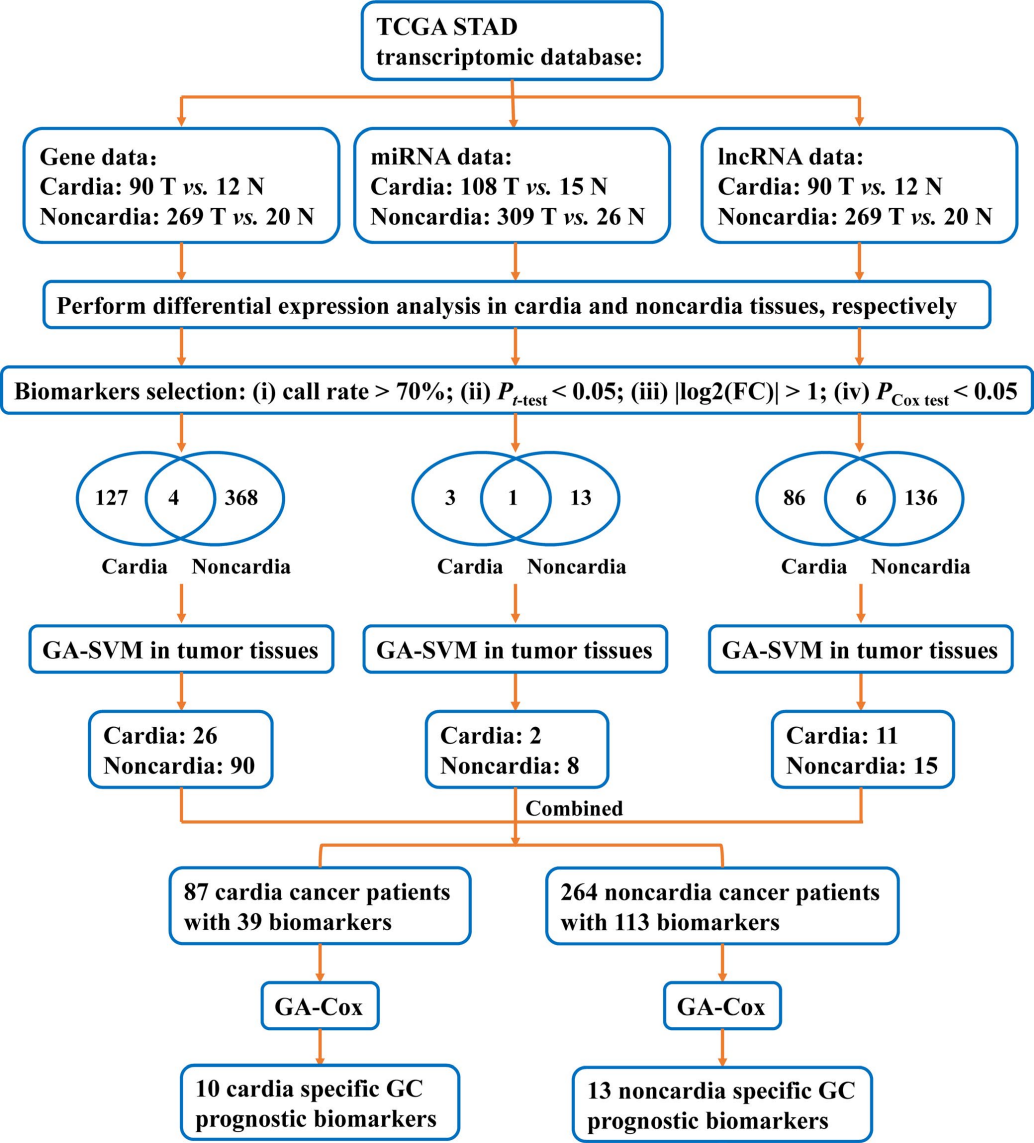
Summary of this study design

To obtain the transcriptomic biomarkers with highest discriminatory power in distinguishing cardia and non‐cardia tumour tissues, we performed GA‐SVM analysis in genes, miRNAs and lncRNAs data sets, respectively (Figure 1). The procedures of GA‐SVM are divided into three steps: (a) GA analysis: perform the GA procedure including selection, crossover and mutation for 200 generations, the fitness is measured using the threefold cross‐validated area under receive operating characteristic (ROC) curve (AUC); (b) variables sort: extract the top 50 variable sets with the highest AUC and calculate each variable's frequency, variables are then sorted by their frequencies in decreasing order; (c) variables selection: construct SVM model with the addition of order‐sorted variables, and obtain a set of variables with the highest threefold cross‐validated AUC (Figure S1).

### 2.4 GA‐Cox and site‐specific risk scores construction

Furthermore, we applied GA‐Cox analysis to identify prognostic factors with highest predictive power in evaluating cardia and non‐cardia GC patients' survival, respectively (Figure 1). Similar to GA‐SVM, the procedures of GA‐Cox are described as follows: (a) GA analysis: perform the GA procedure for 200 generations, the threefold cross‐validated AUC of time‐dependent ROC curve regarding 5‐year survival is used to evaluate the fitness; (b) variables sort: variables identified in the optimal 50 variable sets are sorted by their frequencies in decreasing order; (c) risk score calculation: calculate risk score with the addition of order‐sorted variables, the risk score is defined as: $\sum_{i=1}^{n}\beta_{i}X_{i}$, where *n* is the number of biomarkers, *β_i_* is the Cox regression coefficient for biomarker *i*, and *X_i_* is the expression level (log_2_ transformed) of biomarker *i*; (d) variables selection: construct Cox model using risk score calculated by adding each variable score, and identify a set of site‐specific prognostic factors with the highest threefold cross‐validated AUC (Figure S1).

To evaluate the clinical utility of the site‐specific risk scores calculated by transcriptomic biomarkers in predicting GC survival probability, we used the median of risk score to divide the patients into a high‐ and low‐risk groups among cardia and non‐cardia GC patients. The Kaplan–Meier survival curve and log‐rank test were then applied to compare the survival probability between two groups.

### 2.5 Site‐specific clinical prognostic models construction

We used Cox regression model to perform univariate analysis and multivariate analysis for identifying clinical prognostic factors. After univariate analysis, Cox stepwise regression analysis was used to further screen independent clinical characteristics, with a significance level of *P* < 0.05 for entering and *P* > 0.10 for removing variables. The remaining clinical prognostic factors were used to construct traditional cardia and non‐cardia GC prognostic models.

### 2.6 Site‐specific combined prognostic models construction

We further used site‐specific clinical factors and risk scores to construct combined cardia and non‐cardia GC prognostic model. The predictive power of prognostic model was measured using time‐dependent ROC curve regarding 5‐year survival with R package survivalROC. Besides, the threefold cross‐validation test was used to avoid potential over‐fitting. The difference of ROC curves was evaluated using Wilcoxon rank‐sum test with R package survcomp.

### 2.7 Site‐specific GC nomograms construction

To predict the overall survival probability of cardia and non‐cardia GC patients, the regression coefficients in multivariable Cox regression model were used to generate the nomogram using R package rms, each patient could obtain the total points from the nomogram. The concordance index (C‐index) was then estimated to evaluate the similarity between the actual and predicted survival probability, the larger C‐index (ranges from 0 to 1) indicates a better model performance. The C‐index was also adjusted by bootstrap method with 1000 resamples. Besides, the calibration plot and decision curve were used to evaluate the calibration and clinical utility of the nomograms.

### 2.8 Construction of a user‐friendly webserver

We used R package Shiny (R Core Team, Vienna, Austria) to construct a GC site‐specific molecular system (GC‐SMS), which is freely available and user‐friendly. This system included site‐specific GC molecular databases and survival prediction models. The molecular databases provided the results of differential expression analysis and survival analysis for each biomarker at different GC sites. The survival prediction models could provide the predicted 5‐year survival probability for cardia and non‐cardia GC patients. Total points and corresponding survival probability were calculated by R package nomogramEx (R Core Team, Vienna, Austria).

All analyses were performed using R 3.4.1 software (R Core Team, Vienna, Austria). *P* < 0.05 (two‐side) was statistically significant.

## 3 RESULTS

### 3.1 Basic characteristics of study subjects

The detailed clinical characteristics of 87 cardia and 264 non‐cardia cancer patients are summarized in Table S1. There was a significant difference in neoplasm status (*P* = 0.002) between cardia (28 cases with tumour, 36.36%) and non‐cardia (44 cases with tumour, 19.21%) cancer patients, but the difference of other clinical characteristics (eg sex and age) was not significant (*P* > 0.05).

### 3.2 Identification of prognostic biomarkers

We first used a series of filtering criteria to obtain cardia (including 127 genes, 3 miRNAs and 86 lncRNAs) and non‐cardia (including 368 genes, 13 miRNAs and 136 lncRNAs) specific prognostic biomarkers (Figure 1). GA‐SVM analysis was subsequently used to identify key biomarkers with highest discriminatory power in distinguishing cardia and non‐cardia tumour tissues. Finally, 116 genes (AUC = 0.822; cardia: 26; non‐cardia: 90), 10 miRNAs (AUC = 0.714; cardia: 2; non‐cardia: 8) and 26 lncRNAs (AUC = 0.816; cardia: 11; non‐cardia: 15) were screened for further survival analysis (Figure S2).

**FIGURE 2:**
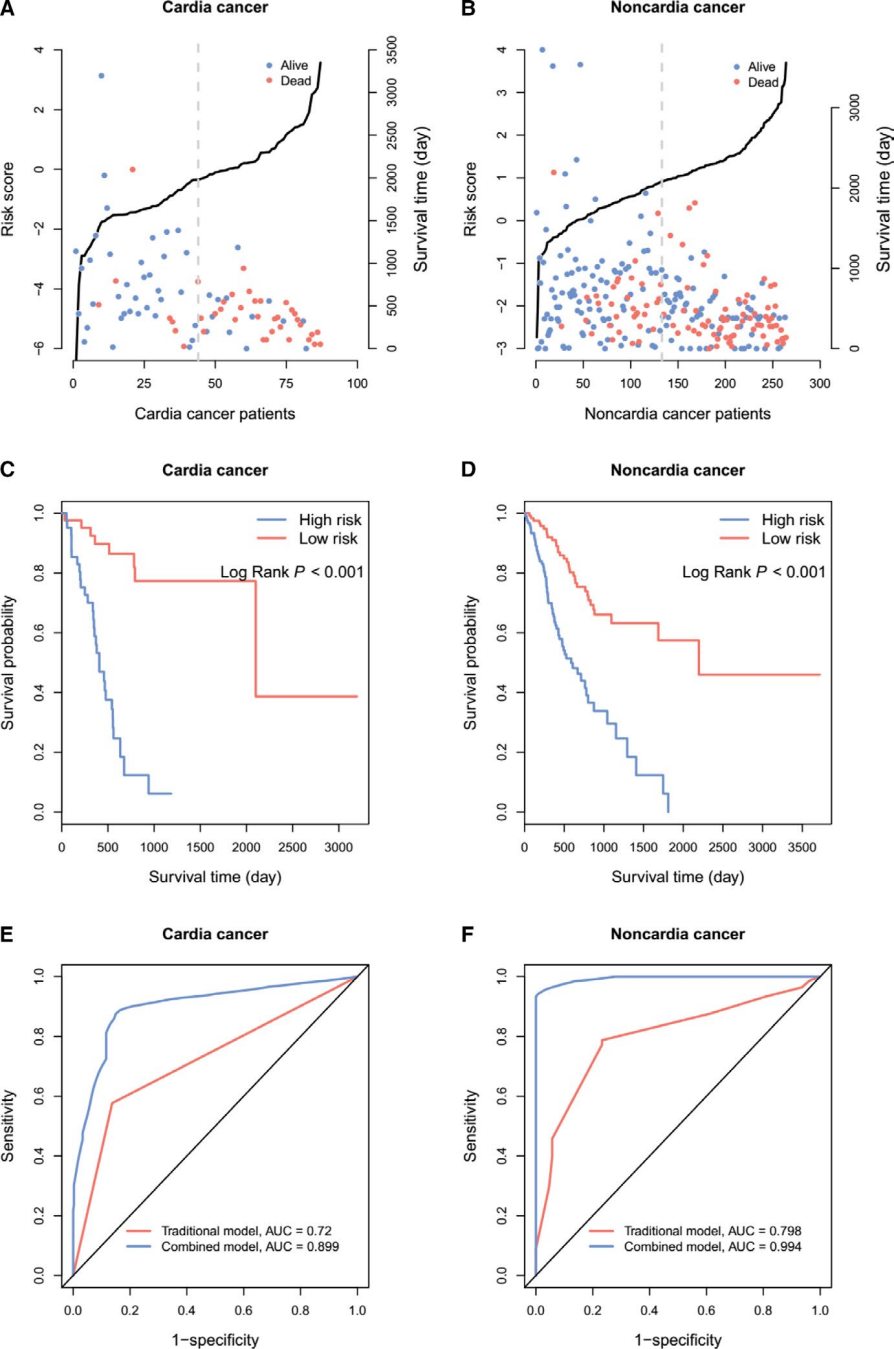
Risk score analysis for 10 and 13 site‐specific biomarkers in cardia and non‐cardia gastric cancer (GC) patients, respectively. A, B, Distribution of site‐specific risk score and survival status in cardia and non‐cardia GC patients. The black lines represented risk scores. The grey dashed lines represented the median value of risk scores. C, D, Kaplan‐Meier survival curves for overall survival outcomes in cardia and non‐cardia GC patients. E, F, The time‐dependent ROC curves regarding 5‐year for cardia and non‐cardia GC prognostic models

Moreover, we applied GA‐Cox analysis to identify key transcriptomic prognostic factors for predicting cardia and non‐cardia GC survival using 39 cardia‐ and 113 non–cardia‐specific biomarkers (Figure 1). For cardia cancer patients, a total of 10 prognostic biomarkers including 7 genes (Table S2) and 3 lncRNAs, with an AUC of 0.913 (Figure S3), were finally identified (Table 1). For non‐cardia cancer patients, we identified 13 prognostic biomarkers including 10 genes (Table S2), 2 miRNAs and 1 lncRNA (AUC = 0.918, Figure S3; Table 2). Furthermore, we divided the patients into high‐ and low‐risk groups using the median of risk score constructed by these biomarkers (Figure 2A,B). Broadly, compared to low‐risk group, high‐risk group had poorer prognosis among cardia and non‐cardia GC patients (log‐rank *P* < 0.001, Figure 2C,D).

**TABLE 1:** Summary of 10 cardia‐specific gastric cancer (GC) prognostic biomarkers

Biomarkers	Prognostic factors	Ensembl ID	Tumour^a^	Normal^a^	FC^b^	*P* ^c^	HR (95% CI)^d^	*P* ^d^
Gene	*TUBB1*	ENSG00000101162.3	0.18	0.07	2.42	4.16E‐04	5.00 (1.32, 18.99)	1.80E‐02
	*APAF1*	ENSG00000120868.12	3.68	1.80	2.04	5.62E‐05	0.50 (0.26, 0.96)	3.72E‐02
	*FAM131B*	ENSG00000159784.16	0.54	0.18	2.99	8.78E‐03	0.19 (0.04, 0.91)	3.72E‐02
	*EN1*	ENSG00000163064.6	0.11	0.02	7.07	2.35E‐04	3.19 (1.37, 7.40)	6.92E‐03
	*TMEM200A*	ENSG00000164484.10	2.14	0.54	4.00	1.34E‐04	1.38 (1.00, 1.90)	4.70E‐02
	*SYT12*	ENSG00000173227.12	0.57	0.15	3.86	4.81E‐03	1.71 (1.05, 2.78)	3.13E‐02
	*WFDC10B*	ENSG00000182931.8	0.39	0.03	11.94	3.86E‐06	2.51 (1.48, 4.25)	6.50E‐04
lncRNA	AC016575.1	ENSG00000272057.1	0.16	0.07	2.26	1.86E‐02	5.47 (1.19, 25.07)	2.87E‐02
	LINC02728	ENSG00000251323.2	0.20	0.07	3.04	6.78E‐03	3.85 (1.57, 9.43)	3.22E‐03
	LINC00412	ENSG00000234772.1	0.16	0.04	4.11	1.76E‐04	0.11 (0.01, 0.93)	4.28E‐02

^a^ Mean value in cardia tissues.

^b^ Fold change, Tumour/Normal.

^c^ *P* value for Student's *t* test in cardia tissues.

^d^ Univariate Cox regression in cardia tissues.

**TABLE 2:** Summary of 13 non–cardia‐specific gastric cancer (GC) prognostic biomarkers

Biomarkers	Prognostic factors	Ensembl ID	Tumour^a^	Normal^a^	FC^b^	*P* ^c^	HR (95% CI)^d^	*P* ^d^
Gene	*CREB3L3*	ENSG00000060566.12	4.67	54.76	0.09	2.06E‐02	1.16 (1.03, 1.31)	1.74E‐02
	*CHADL*	ENSG00000100399.14	1.00	2.13	0.47	3.20E‐07	0.58 (0.38, 0.87)	9.31E‐03
	*LAMP5*	ENSG00000125869.8	2.17	0.77	2.83	2.94E‐04	1.27 (1.05, 1.53)	1.25E‐02
	*CTSV*	ENSG00000136943.9	3.61	0.60	5.99	9.05E‐12	1.22 (1, 1.48)	4.89E‐02
	*CYP19A1*	ENSG00000137869.12	0.12	0.01	11.51	5.52E‐19	3.10 (1.53, 6.25)	1.61E‐03
	*AMDHD1*	ENSG00000139344.6	0.44	0.18	2.45	2.93E‐04	0.54 (0.29, 0.98)	4.25E‐02
	*ALLC*	ENSG00000151360.8	0.06	0.01	5.07	1.64E‐06	3.63 (1.62, 8.12)	1.74E‐03
	*NETO2*	ENSG00000171208.8	1.76	0.28	6.26	1.44E‐19	1.30 (1.02, 1.64)	3.14E‐02
	*HBA2*	ENSG00000188536.11	14.34	41.23	0.35	2.35E‐08	1.16 (1.01, 1.32)	3.04E‐02
	*C5orf58*	ENSG00000234511.7	0.25	0.06	4.39	2.37E‐14	1.91 (1.13, 3.23)	1.55E‐02
miRNA	miR‐7‐2	—	4.37	1.56	2.81	1.09E‐04	0.82 (0.7, 0.98)	2.48E‐02
	miR‐7‐3	—	4.17	1.18	3.53	2.31E‐06	0.83 (0.71, 0.98)	3.21E‐02
lncRNA	LINC01614	ENSG00000230838.1	1.59	0.06	28.20	3.47E‐37	1.36 (1.09, 1.69)	6.32E‐03

^a^ Mean value in non‐cardia tissues.

^b^ Fold change, Tumour/Normal.

^c^ *P* value for Student's *t* test in non‐cardia tissues.

^d^ Univariate Cox regression in non‐cardia tissues.

### 3.3 Construction of site‐specific traditional and combined prognostic models

We initially performed univariate analysis to evaluate the association of each clinical factor with cardia and non‐cardia GC survival (Table S1). We found two risk factors for cardia cancer prognosis, including neoplasm status (HR = 2.75, *P* = 0.005) and residual tumour (HR = 3.59, *P* = 0.007); and four prognostic factors for non‐cardia cancer, including radiation therapy (HR = 0.45, *P* = 0.021), neoplasm status (HR = 3.90, *P* = 1.94E‐09), residual tumour (HR = 3.31, *P* = 8.30E‐05) and tumour stage (stage 3 vs 1*:* HR = 2.24, *P* = 0.036; stage 4 vs 1: HR = 6.73, *P* = 1.82E‐05). Subsequently, we performed a multivariate Cox stepwise regression analysis to select independent clinical factors for constructing cardia and non‐cardia GC traditional prognostic models (Table S3). Finally, neoplasm status (HR = 2.78, *P* = 0.009) was remained in cardia model (AUC = 0.720, Table S4). Non‐cardia model (AUC = 0.798, Table S4) was constructed using radiation therapy (HR = 0.45, *P* = 0.069), neoplasm status (HR = 3.31, *P* < 0.001) and tumour stage (HR = 1.56, *P* = 0.018).

We further introduced the risk scores of 10 cardia and 13 non‐cardia cancer prognostic biomarkers to construct combined site‐specific GC prognostic models, respectively. We found that, with the addition of biomarkers, the combined cardia (AUC = 0.899) and non‐cardia (AUC = 0.994) cancer prognostic models showed stronger predictive power (*P*_cardia_ = 8.75E‐08; *P*_noncardia_ = 7.11E‐16, Table S4, Figure 2E,F) compared to traditional prognostic models. To avoid the potential over‐fitting, the threefold cross‐validation test was used to further confirm the results (cardia: AUC_traditional_ vs AUC_combined_ = 0.726 vs 0.867; non‐cardia: AUC_traditional_ vs AUC_combined_ = 0.797 vs 0.940, Table S4).

### 3.4 Construction of site‐specific nomograms

**FIGURE 3:**
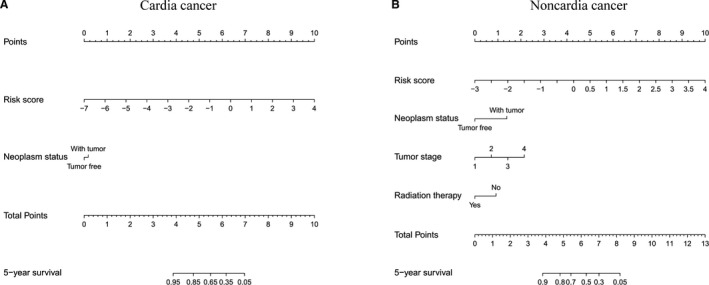
Nomograms that included clinical and transcriptomic prognostic factors to predict the 5‐year overall survival for site‐specific gastric cancer (GC) patients. (A) Cardia cancer; (B) non‐cardia cancer

Furthermore, we constructed two nomograms to show the potential clinical application of the two combined models in cardia (Figure 3A) and non‐cardia (Figure 3B) GC patients' prognosis prediction. Based on the nomograms including multiple prognostic factors, we could predict the 5‐year overall survival probability of cardia and non‐cardia GC patients by drawing a vertical line to the total points. The C‐index for cardia and non‐cardia GC models was 0.816 (95% CI = 0.710‐0.923, adjusted C‐index = 0.811) and 0.812 (95% CI = 0.721‐0.904, adjusted C‐index = 0.801), respectively, revealing the great predictive ability of the two nomogram models. Besides, considering the limited sample size of site‐specific GC patients with follow‐up time over 5 years, we used calibration plots and decision curves regarding 3‐year to evaluate the nomograms, the results also demonstrated the good calibration and clinical application of the two nomograms (Figure S4).

### 3.5 Development of GC‐SMS

An online version of user‐friendly GC site‐specific web server can be accessed at https://njmu‐zhanglab.shinyapps.io/gc_sms/ (Figure S5). Users could perform differential expression analysis and survival analysis for each biomarker simply at different GC sites by clicking the corresponding module (Figure S5A). For example, the user can select a database (gene, miRNA or lncRNA) and a site (overall, cardia or non‐cardia), and input a molecular biomarker (eg *ASB5* for gene, AL627309.1 for lncRNA or miR‐100 for miRNA) to search the results of differential expression analysis and survival analysis. In addition, online implementation of cardia and non‐cardia GC nomogram prognostic models were also available (Figure S5B), predicted 5‐year survival probability can be easily calculated by inputting clinical characteristics and expression value of site‐specific biomarkers.

## 4 DISCUSSION

GC is a heterogeneous tumour with great differences of epidemiologic and clinicopathologic features between cardia cancer and non‐cardia cancer.^12^ For instance, *Helicobacter pylori* infection was demonstrated to be a risk factor for non‐cardia cancer, but not for cardia cancer.^13^ The survival rate of cardia cancer patients was significantly lower than that of non‐cardia cancer patients.^5^ However, few studies were performed to construct site‐specific GC prognostic models to predict the survival probability of cardia and non‐cardia GC patients. In this study, we applied GA‐SVM and GA‐Cox methods to identify 10 cardia‐ and 13 non–cardia‐specific GC prognostic factors, which may be useful for cardia cancer and non‐cardia cancer survival prediction.

With the development of high‐throughput sequence technology, finding accurate biomarkers in high‐dimensional omics data are challenging. GA process, including natural selection, crossover and mutation, is a heuristic algorithm used to explore an optimal solution to a complex problem (such as non‐linear condition).^14^, ^15^, ^16^ Several researchers have applied GA‐based machine learning methods to solve a variety of complex problems in high‐dimensional omics data.^11^, ^17^ Thus, this study proposed two approaches that combine SVM and Cox models with a GA to explore an optimal subset of site‐specific GC prognostic biomarkers. As a result, we finally identified 10 and 13 cardia‐ and non–cardia‐specific GC prognostic factors with a good discriminatory ability, reflecting the GA‐based algorithms’ superior performance.

In the present study, the cardia cancer prognostic model was constructed using 7 genes and 3 lncRNAs; and non‐cardia cancer survival model was constructed using 10 genes, 1 lncRNA and 2 miRNAs. Among these genes, most of them have been demonstrated to be involved in several complex biological processes. For example, *APAF1* is a key apoptosis factor, which is closely related to several cancer‐inducing genes and tumour suppressor genes (eg *p53*).^18^ *EN1* is a transcription factor hypermethylated in multiple cancers, including colorectal cancer, prostate cancer and ovarian cancer, and has been considered as a potential biomarker for several tumours.^19^, ^20^, ^21^ *CYP19A1* has been demonstrated to be associated with the prognosis of GC.^22^ *CREB3L3*, cAMP‐responsive element‐binding protein 3‐like 3, is involved in the inflammatory response.^23^ Several studies have demonstrated the overexpression of *CTSV* in multiple malignant tumours (eg breast ductal carcinoma) and was deemed as a potential prognostic biomarker.^24^ *AMDHD1* has been reported to be overexpressed in adrenal adenoma compared with adrenal carcinoma and is involved in the histidine metabolism pathway.^25^ *NETO2* was reported to be overexpressed in several cancers, including renal cancer, lung cancer and colon cancer.^26^ Hu *et al* also found that high expression of *NETO2* could be considered as a potential biomarker of both advanced tumour progression and poor prognosis in colorectal cancer patients.^27^ In addition to genes, miRNAs are a class of non‐coding RNA molecules that play a vital role in cell differentiation, proliferation and survival by altering the expression of multiple genes.^28^ lncRNAs are a batch of long non‐coding RNA transcripts with a vital role in cancer carcinogenesis and progression.^29^, ^30^ Therefore, we also introduced multiple miRNAs and lncRNAs to the prognostic models for avoiding the limited predictive power of gene sets. In summary, we found that the addition of transcriptomic risk score could improve traditional prognostic models’ predictive accuracy. The discovery of transcriptomic signatures as a prognostic biomarker for cardia cancer and non‐cardia cancer has the potential to be applied in GC risk stratification and personalized therapy.

There were several strengths in this study as follows: (a) this is a comprehensive study to identify the cardia‐ and non–cardia‐specific GC prognostic biomarkers using TCGA STAD transcriptomic data; (b) we proposed GA‐SVM and GA‐Cox methods to explore an optimal subset of prognostic biomarkers; (c) we constructed a combined prognostic model including clinical and transcriptomic prognostic factors, to further improve the predictive power of traditional survival prediction model; and (d) we developed a user‐friendly website to predict the survival probability of cardia and non‐cardia GC patients. In addition, some limitations needed to be noted: (a) the predictive power of transcriptomic prognostic model may be limited, future multi‐omics studies are required to improve the model performance; (b) we need to further validate the two GC prognostic models using external data; and (c) the roles of these biomarkers in influencing site‐specific GC prognosis need to be further validated by biological assays.

In conclusion, based on GA‐SVM and GA‐Cox methods, we identified 23 (cardia: 10 and non‐cardia: 13) site‐specific GC prognostic biomarkers and developed two nomogram prognostic models for predicting cardia cancer and non‐cardia cancer survival, providing more support for the individualized therapy of cardia and non‐cardia GC patients.

## CONFLICTS OF INTEREST

The authors have declared no conflicts of interest.

## AUTHOR CONTRIBUTION

**Junyi Xin:** Conceptualization (equal); Formal analysis (equal); Methodology (equal); Writing‐original draft (equal); Writing‐review & editing (equal). **Yanling Wu:** Formal analysis (equal); Methodology (equal); Writing‐review & editing (equal). **Xiaowei Wang:** Formal analysis (equal); Writing‐review & editing (equal). **Shuwei Li:** Data curation (equal); Resources (equal). **Haiyan Chu:** Data curation (equal); Resources (equal). **Meilin Wang:** Conceptualization (equal); Investigation (equal); Writing‐review & editing (equal). **Mulong Du:** Conceptualization (equal); Investigation (equal); Project administration (equal); Supervision (equal); Writing‐review & editing (equal). **Zhengdong Zhang:** Conceptualization (equal); Funding acquisition (equal); Project administration (equal); Supervision (equal); Writing‐review & editing (equal).

## Supporting information

Appendix S1

## Data Availability

All data will be made available upon request.
