# Supplementary material for: A transcriptomic study for identifying cardia‐ and non–cardia‐specific gastric cancer prognostic factors using genetic algorithm‐based methods
Source: J Cell Mol Med. 2020 Jul 10;24(16):9457–65. doi: 10.1111/jcmm.15618 (PMC7417703; doi:10.1111/jcmm.15618)
Supplement: Supplementary file 1 — Appendix S1 [file JCMM-24-9457-s001.docx]

**Supplementary Table 1**.Clinical characteristics of cardia and noncardia gastric cancer (GC) patients

| Clinical variables | Cardia (n=87) | | | Noncardia (n = 264) | | | *P*^a^ |
| --- | --- | --- | --- | --- | --- | --- | --- |
|  | N | HR (95% CI) | *P* | N | HR (95% CI) | *P* |  |
| Sex |  |  |  |  |  |  | 0.096 |
| Male | 62 | 1.77 (0.81, 3.88) | 0.153 | 162 | 1.08 (0.72, 1.64) | 0.699 |  |
| Female | 25 | 1 |  | 102 | 1 |  |  |
| Age |  |  |  |  |  |  |  |
| ≤ 60 | 28 | 1 |  | 87 | 1 |  | 0.860 |
| > 60 | 59 | 1.5 (0.74, 3.06) | 0.265 | 175 | 1.48 (0.94, 2.32) | 0.088 |  |
| Family history of stomach cancer |  |  |  |  |  |  | 0.855 |
| No | 59 | 1 |  | 201 | 1 |  |  |
| Yes | 4 | 1.09 (0.33, 3.58) | 0.892 | 10 | 0.91 (0.37, 2.27) | 0.847 |  |
| Neoplasm status |  |  |  |  |  |  | 0.002 |
| With tumor | 28 | 2.75 (1.36, 5.56) | 0.005 | 44 | 3.90 (2.5, 6.07) | 1.94E-09 |  |
| Tumor free | 49 | 1 |  | 185 | 1 |  |  |
| Hpylori infection |  |  |  |  |  |  | 0.102 |
| No | 37 | 1 |  | 98 | 1 |  |  |
| Yes | 1 | 3.02 (0.39, 23.62) | 0.293 | 16 | 0.38 (0.13, 1.08) | 0.070 |  |
| Residual tumor |  |  |  |  |  |  | 0.485 |
| No (R0) | 70 | 1 |  | 215 | 1 |  |  |
| Yes (R1/R2) | 8 | 3.59 (1.41, 9.1) | 0.007 | 18 | 3.31 (1.82, 6.02) | 8.30E-05 |  |
| Radiation therapy |  |  |  |  |  |  | 0.492 |
| No | 32 | 1 |  | 105 | 1 |  |  |
| Yes | 12 | 0.47 (0.15, 1.44) | 0.187 | 30 | 0.45 (0.23, 0.88) | 0.021 |  |
| Tumor stage |  |  |  |  |  |  | 0.393 |
| 1 | 13 | 1 |  | 33 | 1 |  |  |
| 2 | 28 | 1.51 (0.41, 5.53) | 0.531 | 76 | 1.47 (0.65, 3.35) | 0.359 |  |
| 3 | 29 | 2.34 (0.68, 8.02) | 0.178 | 117 | 2.24 (1.06, 4.75) | 0.036 |  |
| 4 | 10 | 1.72 (0.40, 7.33) | 0.464 | 23 | 6.73 (2.81, 16.10) | 1.82E-05 |  |

^a^ Chi-square test or fisher’s exact test between cardia and noncardia GC patients.

**Supplementary Table 2**. Molecular functions of site-specific gastric cancer (GC) prognostic genes

| Site | Genes | Ensembl id (GRCh38) | Molecular function^a^ |
| --- | --- | --- | --- |
| Cardia | *TUBB1* | ENSG00000101162.3 | UniProtKB/Swiss-Prot Function: Tubulin is the major constituent of microtubules. It binds two moles of GTP, one at an exchangeable site on the beta chain and one at a non-exchangeable site on the alpha chain (By similarity). |
|  | *APAF1* | ENSG00000120868.12 | UniProtKB/Swiss-Prot Function: Oligomeric Apaf-1 mediates the cytochrome c-dependent autocatalytic activation of pro-caspase-9 (Apaf-3), leading to the activation of caspase-3 and apoptosis. This activation requires ATP. Isoform 6 is less effective in inducing apoptosis.  UniProtKB/Swiss-Prot Induction: By E2F and p53/TP53 in apoptotic neurons (PubMed:11389439). Tranlation is inhibited by HNRPA1, which binds to the IRES of APAF1 mRNAs (PubMed:31498791).  GENATLAS Biochemistry: cytoplasmic scaffolding apoptotic protease activating factor 1,89kDa,forming a ternary complex ATP,datp dependent with cytochrome C1 and the initiator of apoptosis caspase 9,not inhibited by binding to BCL2 |
|  | *FAM131B* | ENSG00000159784.16 | - |
|  | *EN1* | ENSG00000163064.6 | GENATLAS Biochemistry: Drosophila segment-polarity gene engrailed homolog 1,homeo domain encoding gene,expressed in embryonic ventral limb ectoderm,regulator of the ventral limb patterning,inhibits WNT7A expression in ventral limb bud |
|  | *TMEM200A* | ENSG00000164484.10 | - |
|  | *SYT12* | ENSG00000173227.12 | UniProtKB/Swiss-Prot Function: Synaptic vesicle phosphoprotein that enhances spontaneous neurotransmitter release but does not effect induced neurotransmitter release (By similarity). Unlike other synaptotagmins, it does not bind Ca(2+) or phospholipids (By similarity). Essential for mossy-fiber long-term potentiation in the hippocampus (By similarity). |
|  | *WFDC10B* | ENSG00000182931.8 | - |
| Noncardia | *CREB3L3* | ENSG00000060566.12 | UniProtKB/Swiss-Prot Function: Transcription factor that may act during endoplasmic reticulum stress by activating unfolded protein response target genes. Activated in response to cAMP stimulation. In vitro, binds to the cAMP response element (CRE) and box-B element. Activates transcription through box-B element. Activates transcription through CRE (By similarity). Seems to function synergistically with ATF6. In acute inflammatory response, may activate expression of acute phase response (APR) genes. May be involved in growth suppression. |
|  | *CHADL* | ENSG00000100399.14 | UniProtKB/Swiss-Prot Function: Potential negative modulator of chondrocyte differentiation. Inhibits collagen fibrillogenesis in vitro. May influence chondrocyte's differentiation by acting on its cellular collagenous microenvironment. |
|  | *LAMP5* | ENSG00000125869.8 | UniProtKB/Swiss-Prot Function: Plays a role in short-term synaptic plasticity in a subset of GABAergic neurons in the brain.  UniProtKB/Swiss-Prot Induction: Up-regulated upon CpG dinucleotides activation. Down-regulated upon activation by Toll-like receptor (TLR) ligands.  GENATLAS Biochemistry: lysosomal-associated multispanning membrane protein,preferentially expressed in hematopoietic cell |
|  | *CTSV* | ENSG00000136943.9 | Cysteine protease. May have an important role in corneal physiology.  UniProtKB/Swiss-Prot CatalyticActivity: Reaction=The recombinant enzyme hydrolyzes proteins (serum albumin, collagen) and synthetic substrates (Z-Phe-Arg-NHMec > Z-Leu-Arg-NHMec > Z-Val-Arg-NHMec).; EC=3.4.22.43; Evidence=. ;.  GENATLAS Biochemistry: cathepsin V,thymus and testis specific cysteine protease |
|  | *CYP19A1* | ENSG00000137869.12 | UniProtKB/Swiss-Prot Function: A cytochrome P450 monooxygenase that catalyzes the conversion of C19 androgens, androst-4-ene-3,17-dione (androstenedione) and testosterone to the C18 estrogens, estrone and estradiol, respectively (PubMed:27702664, PubMed:2848247). Catalyzes three successive oxidations of C19 androgens: two conventional oxidations at C19 yielding 19-hydroxy and 19-oxo/19-aldehyde derivatives, followed by a third oxidative aromatization step that involves C1-beta hydrogen abstraction combined with cleavage of the C10-C19 bond to yield a phenolic A ring and formic acid (PubMed:20385561). Alternatively, the third oxidative reaction yields a 19-norsteroid and formic acid. Converts dihydrotestosterone to delta1,10-dehydro 19-nordihydrotestosterone and may play a role in homeostasis of this potent androgen (PubMed:22773874). Also displays 2-hydroxylase activity toward estrone (PubMed:22773874). Mechanistically, uses molecular oxygen inserting one oxygen atom into a substrate, and reducing the second into a water molecule, with two electrons provided by NADPH via cytochrome P450 reductase (CPR; NADPH-ferrihemoprotein reductase) (PubMed:20385561, PubMed:22773874).  UniProtKB/Swiss-Prot CatalyticActivity: Reaction=3 O2 + 3 reduced [NADPH--hemoprotein reductase] + testosterone = 17beta-estradiol + formate + 4 H(+) + 4 H2O + 3 oxidized [NADPH--hemoprotein reductase]; Xref=Rhea:RHEA:38191, Rhea:RHEA-COMP:11964, Rhea:RHEA-COMP:11965, ChEBI:CHEBI:15377, ChEBI:CHEBI:15378, ChEBI:CHEBI:15379, ChEBI:CHEBI:15740, ChEBI:CHEBI:16469, ChEBI:CHEBI:17347, ChEBI:CHEBI:57618, ChEBI:CHEBI:58210; EC=1.14.14.14; Evidence=. ; PhysiologicalDirection=left-to-right; Xref=Rhea:RHEA:38192; Evidence=. ;.  UniProtKB/Swiss-Prot CatalyticActivity: Reaction=androst-4-ene-3,17-dione + 3 O2 + 3 reduced [NADPH--hemoprotein reductase] = estrone + formate + 4 H(+) + 4 H2O + 3 oxidized [NADPH--hemoprotein reductase]; Xref=Rhea:RHEA:38195, Rhea:RHEA-COMP:11964, Rhea:RHEA-COMP:11965, ChEBI:CHEBI:15377, ChEBI:CHEBI:15378, ChEBI:CHEBI:15379, ChEBI:CHEBI:15740, ChEBI:CHEBI:16422, ChEBI:CHEBI:17263, ChEBI:CHEBI:57618, ChEBI:CHEBI:58210; EC=1.14.14.14; Evidence=. ; PhysiologicalDirection=left-to-right; Xref=Rhea:RHEA:38196; Evidence=. ;. CP19A_HUMAN,P11511 UniProtKB/Swiss-Prot CatalyticActivity: Reaction=androst-4-ene-3,17-dione + O2 + reduced [NADPH--hemoprotein reductase] = 19-hydroxyandrost-4-ene-3,17-dione + H(+) + H2O + oxidized [NADPH--hemoprotein reductase]; Xref=Rhea:RHEA:38199, Rhea:RHEA-COMP:11964, Rhea:RHEA-COMP:11965, ChEBI:CHEBI:15377, ChEBI:CHEBI:15378, ChEBI:CHEBI:15379, ChEBI:CHEBI:16422, ChEBI:CHEBI:27576, ChEBI:CHEBI:57618, ChEBI:CHEBI:58210; Evidence=. ; PhysiologicalDirection=left-to-right; Xref=Rhea:RHEA:38200; Evidence=. ;.  UniProtKB/Swiss-Prot CatalyticActivity: Reaction=19-hydroxyandrost-4-ene-3,17-dione + O2 + reduced [NADPH--hemoprotein reductase] = 19-oxo-androst-4-ene-3,17-dione + H(+) + 2 H2O + oxidized [NADPH--hemoprotein reductase]; Xref=Rhea:RHEA:38203, Rhea:RHEA-COMP:11964, Rhea:RHEA-COMP:11965, ChEBI:CHEBI:799, ChEBI:CHEBI:15377, ChEBI:CHEBI:15378, ChEBI:CHEBI:15379, ChEBI:CHEBI:27576, ChEBI:CHEBI:57618, ChEBI:CHEBI:58210; Evidence=. ; PhysiologicalDirection=left-to-right; Xref=Rhea:RHEA:38204; Evidence=. ;.  UniProtKB/Swiss-Prot CatalyticActivity: Reaction=19-oxo-androst-4-ene-3,17-dione + O2 + reduced [NADPH--hemoprotein reductase] = estrone + formate + 2 H(+) + H2O + oxidized [NADPH--hemoprotein reductase]; Xref=Rhea:RHEA:38207, Rhea:RHEA-COMP:11964, Rhea:RHEA-COMP:11965, ChEBI:CHEBI:799, ChEBI:CHEBI:15377, ChEBI:CHEBI:15378, ChEBI:CHEBI:15379, ChEBI:CHEBI:15740, ChEBI:CHEBI:17263, ChEBI:CHEBI:57618, ChEBI:CHEBI:58210; Evidence=. ; PhysiologicalDirection=left-to-right; Xref=Rhea:RHEA:38208; Evidence=. ;. UniProtKB/Swiss-Prot CatalyticActivity: Reaction=estrone + O2 + reduced [NADPH--hemoprotein reductase] = 2-hydroxyestrone + H(+) + H2O + oxidized [NADPH--hemoprotein reductase]; Xref=Rhea:RHEA:47208, Rhea:RHEA-COMP:11964, Rhea:RHEA-COMP:11965, ChEBI:CHEBI:1156, ChEBI:CHEBI:15377, ChEBI:CHEBI:15378, ChEBI:CHEBI:15379, ChEBI:CHEBI:17263, ChEBI:CHEBI:57618, ChEBI:CHEBI:58210; Evidence=. ; PhysiologicalDirection=left-to-right; Xref=Rhea:RHEA:47209; Evidence=. ;.  UniProtKB/Swiss-Prot CatalyticActivity: Reaction=17beta-hydroxy-5alpha-androstan-3-one + O2 + reduced [NADPH--hemoprotein reductase] = 17beta,19-dihydroxy-3-oxo-5alpha-androstanone + H(+) + H2O + oxidized [NADPH--hemoprotein reductase]; Xref=Rhea:RHEA:53200, Rhea:RHEA-COMP:11964, Rhea:RHEA-COMP:11965, ChEBI:CHEBI:15377, ChEBI:CHEBI:15378, ChEBI:CHEBI:15379, ChEBI:CHEBI:16330, ChEBI:CHEBI:57618, ChEBI:CHEBI:58210, ChEBI:CHEBI:137031; Evidence=. ; PhysiologicalDirection=left-to-right; Xref=Rhea:RHEA:53201; Evidence=. ;.  UniProtKB/Swiss-Prot CatalyticActivity: Reaction=17beta,19-dihydroxy-3-oxo-5alpha-androstanone + O2 + reduced [NADPH--hemoprotein reductase] = 17beta-hydroxy-3,19-dioxo-5alpha-androstanone + H(+) + 2 H2O + oxidized [NADPH--hemoprotein reductase]; Xref=Rhea:RHEA:53204, Rhea:RHEA-COMP:11964, Rhea:RHEA-COMP:11965, ChEBI:CHEBI:15377, ChEBI:CHEBI:15378, ChEBI:CHEBI:15379, ChEBI:CHEBI:57618, ChEBI:CHEBI:58210, ChEBI:CHEBI:137031, ChEBI:CHEBI:137032; Evidence=. ; PhysiologicalDirection=left-to-right; Xref=Rhea:RHEA:53205; Evidence=. ;.  UniProtKB/Swiss-Prot CatalyticActivity: Reaction=17beta-hydroxy-3,19-dioxo-5alpha-androstanone + O2 + reduced [NADPH--hemoprotein reductase] = 17beta-hydroxy-3-oxo-19-nor-5alpha-androst-1-ene + formate + 2 H(+) + H2O + oxidized [NADPH--hemoprotein reductase]; Xref=Rhea:RHEA:53276, Rhea:RHEA-COMP:11964, Rhea:RHEA-COMP:11965, ChEBI:CHEBI:15377, ChEBI:CHEBI:15378, ChEBI:CHEBI:15379, ChEBI:CHEBI:15740, ChEBI:CHEBI:57618, ChEBI:CHEBI:58210, ChEBI:CHEBI:137032, ChEBI:CHEBI:137110; Evidence=. ; PhysiologicalDirection=left-to-right; Xref=Rhea:RHEA:53277; Evidence=. ;.  UniProtKB/Swiss-Prot BiophysicochemicalProperties: Kinetic parameters: KM=0.46 uM for androst-4-ene-3,17-dione {ECO:0000269 PubMed:27702664}; KM=0.044 uM for androst-4-ene-3,17-dione (19-hydroxylation) {ECO:0000269 PubMed:20385561}; KM=21 uM for 19-hydroxyandrost-4-ene-3,17-dione {ECO:0000269 PubMed:20385561}; KM=18 uM for 19-oxo-androst-4-ene-3,17-dione {ECO:0000269 PubMed:20385561}; KM=2.7 uM for estrone (2-hydroxylation) {ECO:0000269 PubMed:22773874}; KM=3.8 uM for 17beta-hydroxy-5alpha-androstan-3-one (19- hydroxylation) {ECO:0000269 PubMed:22773874}; KM=3.2 uM for 17beta,19-dihydroxy-3-oxo-5alpha-androstanone {ECO:0000269 PubMed:22773874}; KM=7.6 uM for 17beta-hydroxy-3,19-dioxo-5alpha-androstanone {ECO:0000269 PubMed:22773874}; Note=kcat is 0.060 sec(-1) with androst-4-ene-3,17-dione as substrate (PubMed:20385561). kcat is 0.13 sec(-1) with 19-oxo- androst-4-ene-3,17-dione (PubMed:20385561). kcat is 0.42 sec(-1) with androst-4-ene-3,17-dione as substrate (PubMed:20385561). kcat is 0.046 min(-1) with estrone as substrate (PubMed:22773874). kcat is 0.27 min(-1) with 17beta-hydroxy- 5alpha-androstan-3-one as substrate (PubMed:22773874). kcat is 0.32 min(-1) with 17beta,19-dihydroxy-3-oxo-5alpha-androstanone as substrate (PubMed:22773874). kcat is 0.77 min(-1) with 17beta-hydroxy-3,19-dioxo-5alpha-androstanone as substrate (PubMed:22773874). |
|  | *AMDHD1* | ENSG00000139344.6 | UniProtKB/Swiss-Prot CatalyticActivity: Reaction=4-imidazolone-5-propanoate + H2O = N-formimidoyl-L-glutamate; Xref=Rhea:RHEA:23660, ChEBI:CHEBI:15377, ChEBI:CHEBI:58928, ChEBI:CHEBI:77893; EC=3.5.2.7;. |
|  | *ALLC* | ENSG00000151360.8 | UniProtKB/Swiss-Prot Function: The function of this enzyme is unclear as allantoicase activity is not known to exist in mammals. ALLC_HUMAN,Q8N6M5 UniProtKB/Swiss-Prot CatalyticActivity: Reaction=allantoate + H2O = (S)-ureidoglycolate + urea; Xref=Rhea:RHEA:11016, ChEBI:CHEBI:15377, ChEBI:CHEBI:16199, ChEBI:CHEBI:17536, ChEBI:CHEBI:57296; EC=3.5.3.4;. |
|  | *NETO2* | ENSG00000171208.8 | UniProtKB/Swiss-Prot Function: Accessory subunit of neuronal kainate-sensitive glutamate receptors, GRIK2 and GRIK3. Increases kainate-receptor channel activity, slowing the decay kinetics of the receptors, without affecting their expression at the cell surface, and increasing the open probability of the receptor channels. Modulates the agonist sensitivity of kainate receptors. Slows the decay of kainate receptor-mediated excitatory postsynaptic currents (EPSCs), thus directly influencing synaptic transmission (By similarity). |
|  | *HBA2* | ENSG00000188536.11 | UniProtKB/Swiss-Prot Function: Involved in oxygen transport from the lung to the various peripheral tissues. HBA_HUMAN,P69905 GENATLAS Biochemistry: hemoglobin,alpha 2,adult |
|  | *C5orf58* | ENSG00000234511.7 | - |

^a^ Annotated by GeneCards (<https://www.genecards.org/>).

**Supplementary Table 3**. Multivariate Cox regression of clinical characteristics for cardia cancer and noncardia cancer

| Site | Clinical variables | HR (95% CI) | *P* |
| --- | --- | --- | --- |
| Cardia | Neoplasm status | 2.78 (1.30, 5.96) | 0.009 |
| Noncardia | Radiation therapy | 0.45 (0.19, 1.06) | 0.069 |
|  | Neoplasm status | 3.31 (1.70, 6.44) | < 0.001 |
|  | Tumor stage (stage 4 *vs.* 3 *vs.* 2 *vs.* 1) | 1.56 (1.04, 2.33) | 0.018 |

**Supplementary Table 4**. Discriminatory ability of traditional and combined prognostic models for cardia cancer and noncardia cancer

| Site | Models | AUC | *P* | 3-fold cross-validated AUC | C-index | Adjusted C-index^c^ |
| --- | --- | --- | --- | --- | --- | --- |
| Cardia^a^ | Traditional | 0.720 | - | 0.726 | 0.593 (0.504, 0.683) | 0.591 |
|  | Combined | 0.899 | 8.75E-08 | 0.867 | 0.816 (0.710, 0.923) | 0.811 |
| Noncardia^b^ | Traditional | 0.798 | - | 0.797 | 0.744 (0.654, 0.834) | 0.735 |
|  | Combined | 0.994 | 7.11E-16 | 0.940 | 0.812 (0.721, 0.904) | 0.801 |

^a^ Traditional model included Neoplasm status; combined model included Neoplasm status and prognostic risk score.

^b^ Traditional model included neoplasm status, radiation therapy and tumor stage; combined model included neoplasm status, radiation therapy, tumor stage and prognostic risk score.

^c^ Bootstrap method.


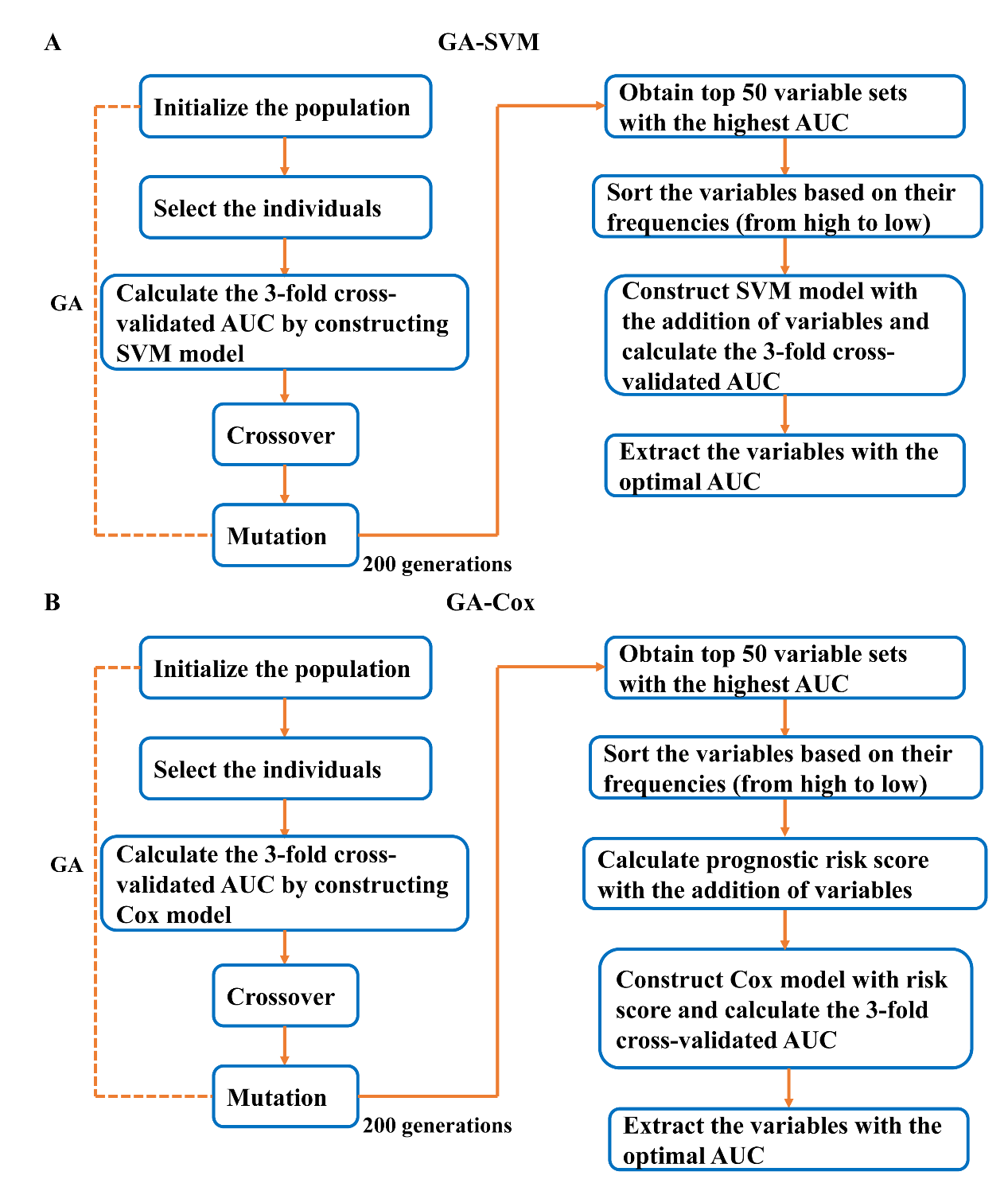


**Supplementary Figure 1**. Summary of the genetic algorithm (GA)-based support vector machine (GA-SVM) and GA-based Cox regression method (GA-Cox) procedures. (A) GA-SVM; (B) GA-Cox.


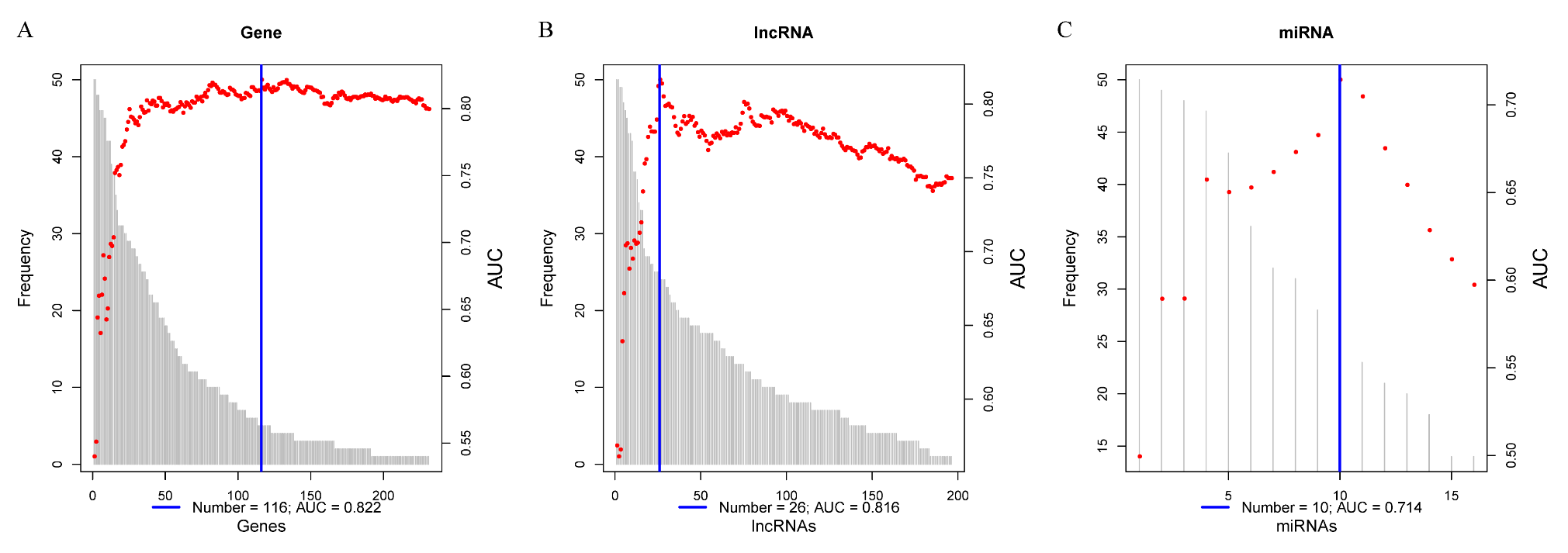


**Supplementary Figure 2**. Genetic algorithm (GA)-based support vector machine (GA-SVM) analysis in gastric cancer (GC) tumor tissues. (A) Genes; (B) lncRNAs; (C) miRNAs.


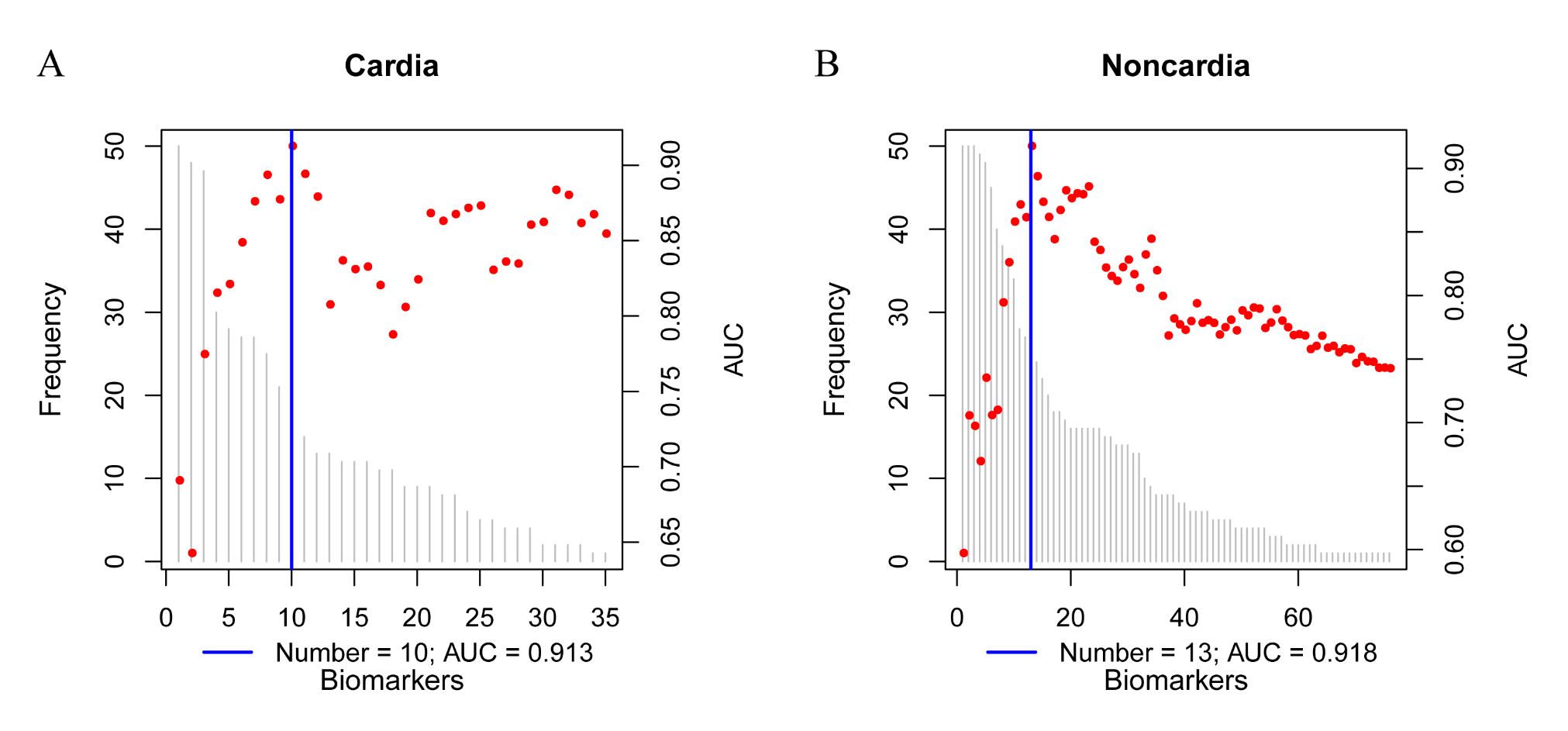


**Supplementary Figure 3**. Genetic algorithm (GA)-based Cox regression method (GA-Cox) analysis in gastric cancer (GC) tumor tissues. (A) Cardia cancer; (B) noncardia cancer.


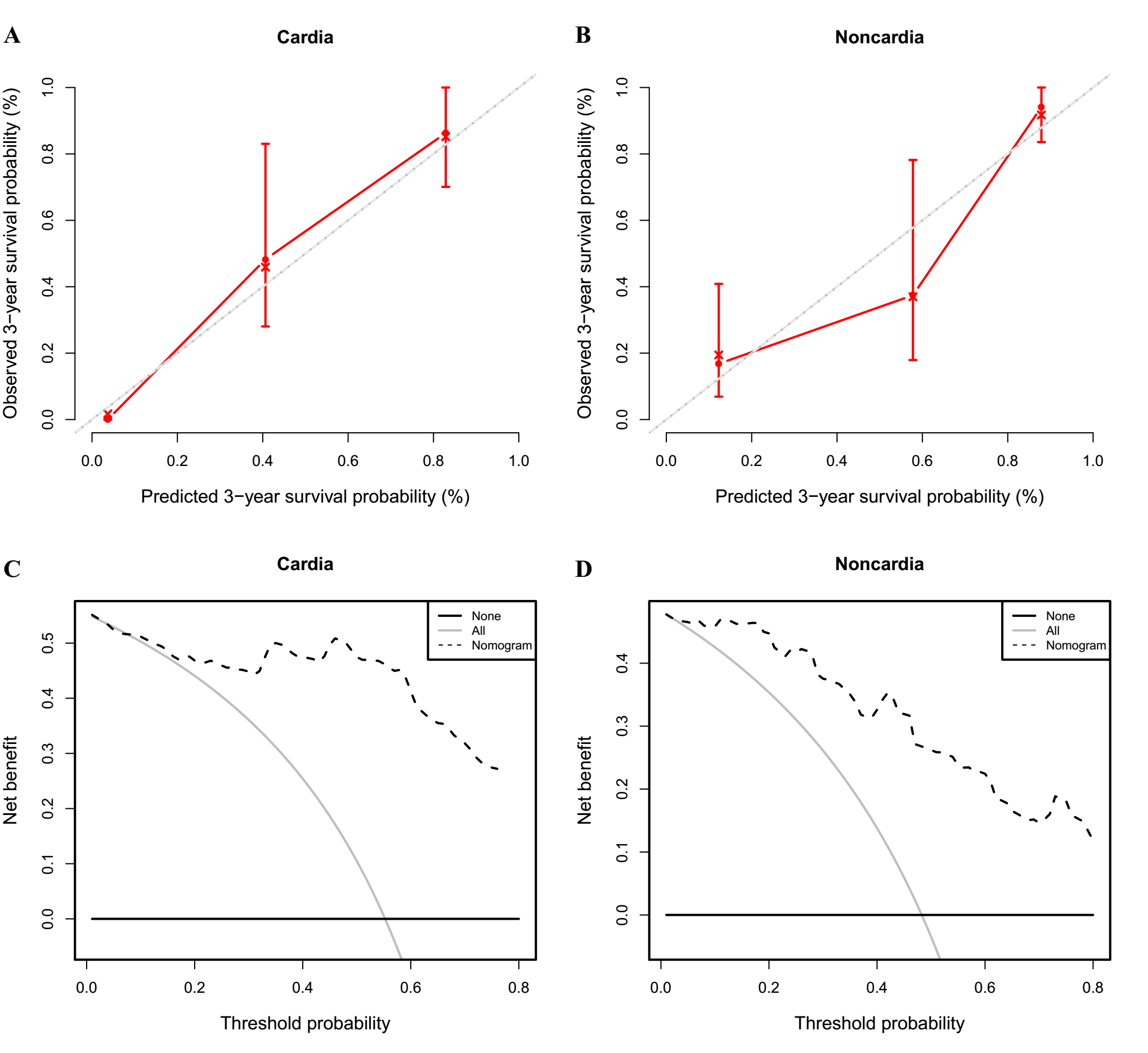


**Supplementary Figure 4**. Calibration plots and decision curves for cardia and noncardia gastric cancer (GC) nomograms regarding 3-year. (A-B) Calibration curves for cardia cancer and noncardia cancer; (C-D) decision curves for cardia cancer and noncardia cancer.


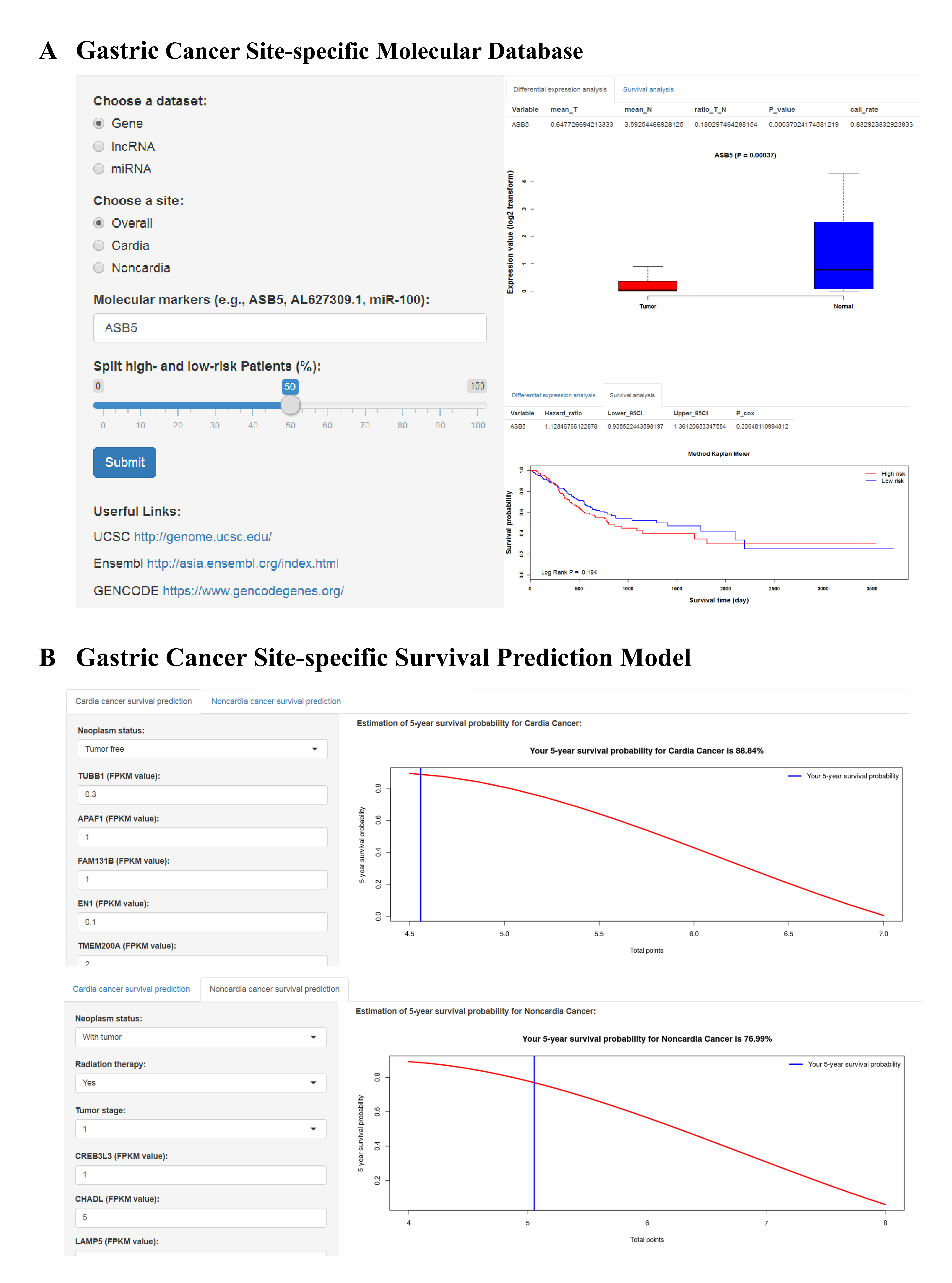


**Supplementary Figure 5.** Overview of Gastric Cancer (GC) Site-specific Molecular System (GC-SMS, <https://njmu-zhanglab.shinyapps.io/gc_sms/>). (A) GC site-specific molecular database; (B) GC site-specific survival prediction model.
